# Prediction kinetic, energy and exergy of quince under hot air dryer using ANNs and ANFIS

**DOI:** 10.1002/fsn3.1347

**Published:** 2019-12-12

**Authors:** Yousef Abbaspour‐Gilandeh, Ahmad Jahanbakhshi, Mohammad Kaveh

**Affiliations:** ^1^ Department of Biosystems Engineering College of Agriculture and Natural Resources University of Mohaghegh Ardabili Ardabil Iran

**Keywords:** adaptive neuro‐fuzzy inference system, artificial neural networks, drying, quince, thermodynamic parameters

## Abstract

This study aimed to predict the drying kinetics, energy utilization (*E_u_*), energy utilization ratio (*EUR*), exergy loss, and exergy efficiency of quince slice in a hot air (HA) dryer using artificial neural networks and ANFIS. The experiments were performed at air temperatures of 50, 60, and 70°C and air velocities of 0.6, 1.2, and 1.8 m/s. The thermal parameters were determined using thermodynamic relations. Increasing air temperature and air velocity increased the effective moisture diffusivity (*D_eff_*), *E_u_*, *EUR*, exergy efficiency, and exergy loss. The value of the *D_eff_* was varied from 4.19 × 10^–10^ to 1.18 × 10^–9^ m^2^/s. The highest value *E_u_*, *EUR,* and exergy loss and exergy efficiency were calculated 0.0694 kJ/s, 0.882, 0.044 kJ/s, and 0.879, respectively. Midilli et al. model, ANNs, and ANFIS model, with a determination coefficient (*R* ^2^) of .9992, .9993, and .9997, provided the best performance for predicting the moisture ratio of quince fruit. Also, the ANFIS model, in comparison with the artificial neural networks model, was better able to predict *E_u_*, *EUR*, exergy efficiency, and exergy loss, with *R* ^2^ of .9989, .9988, .9986, and .9978, respectively.

## Graphical abstract

In this study, drying kinetics, effective moisture diffusivity (Deff), activation energy (Ea), specific energy consumption (SEC), energy utilization (Eu), energy utilization ratio (EUR), exergy loss, and exergy efficiency in a hot air (HA) dryer are presented for quince fruit slices. Midilli et al. model, ANNs, and ANFIS model, with a determination coefficient (*R* ^2^) of .9992, .9993, and 0.9997 provided the best performance for predicting the moisture ratio of quince fruit. Also, the ANFIS model in comparison with the artificial neural network model was better able to predict Eu, EUR, exergy efficiency, and exergy loss, with the *R* ^2^ of .9989, .9988, .9986, and .9978, respectively.

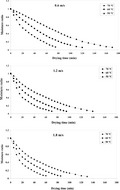

## 1 INTRODUCTION

Drying is one of the oldest procedures for preserving food and agricultural products. Drying is defined as the reduction in moisture from the products and is the most important process for preserving agricultural products since it has a significant effect on the quality of the dried products. The principle objective in drying agricultural products is the reduction in the moisture content to a level that allows safe storage over an extended period (Mohammadi, Tabatabaekoloor, & Motevali, 2019). Nevertheless, the high consumption of energy in the food drying industry has made it the most consuming and the most important industrial operation. Therefore, one of the most substantial challenges in dried fruit industry is to reduce the cost of energy sources to produce dry quality products. So, useful thermodynamic analysis of dryers is necessary when we aim to save energy consumption and optimize process variables (Roknul Azam, Zhang, Law, & Mujumdar, 2019; Shende & Datta, 2019).

Energy and exergy analysis is applied to determine energy needed to dry the product and exergy loss at each stage of the process. Exergy is defined as the maximum amount of work, which can be produced by a stream of matter, heat, or work as it comes to equilibrium with a reference environment. In the drying industry, the goal is to use a minimum amount of energy for maximum moisture removal for the desired final conditions of the product. So, focusing on energy and exergy analysis is very important (Lingayat, Chandramohan, & Raju, 2018; Yogendrasasidhar & Setty, 2018).

Şevik, Aktaş, Dolgun, Arslan, and Tuncer (2019) analyzed the exergy and energy in the process of drying mint and apple slices in a solar and solar‐infrared. The results indicated that the loss of exergy and exergy efficiency increases by increasing the air temperature. Exergy efficiency for mint in solar dryer and solar‐infrared was 69.35% and 59.07%, respectively. Akpinar, Midilli, and Bicer (2006) analyzed the energy and exergy in pumpkin. They reported that the pumpkin dried within the time range of 5.66–12 hr with a loss of exergy from 0 to 1.165 kJ/s. The maximum exergy of the system input was 2.198 kJ/s. Also, with increased exergy loss, the energy used in the solar dryer increased. Karthikeyan and Murugavelh (2018) studied the energy and exergy required to dry turmeric in a mixed‐mode forced convection solar tunnel dryer and concluded that the loss of exergy and energy utilization ratio and its efficiency was increased with increasing temperature.

Recently, several studies have been carried out to energy and exergy analysis in the process of drying products such as potato slices in solar dryer (Kesavan, Arjunan, & Vijayan, 2019), turmeric slices in microwave dryer (Surendhar, Sivasubramanian, Vidhyeswari, & Deepanraj, 2019), Kodo millet grains and fenugreek seeds using wall heated fluidized bed dryer (Yogendrasasidhar & Setty, 2018), dog‐rose flowers with a hybrid infrared‐hot air dryer (Motevali, Jafari, & Hashemi, 2018), and tomato slices in a solar dryer (Arepally, Ravula, Malik, & Kamidi, 2017).

The base of intelligent methods work is using hidden knowledge in the experimental data, trying to extract the inherent relationships among them and generalizing results to other situations. Artificial neural networks are one of the most essential methods used in the field of artificial intelligence was inspired by how the human brain works, training takes place first, and then the information related to the data is stored in the form of the network's weights (Jahanbakhshi, Ghamari, & Heidarbeigi, 2017; Sun, Zhang, & Mujumdar, 2019). Artificial neural networks and ANFIS have been successful in estimations in natural processes. These methods have advantages over many conventional statistical and deterministic procedures. Compared to linear regression models, they do not necessitate the placement of prediction values around the mean and thus reflect the real data variability (Jahanbakhshi & Salehi, 2019; Kaveh, Jahanbakhshi, Abbaspour‐Gilandeh, Taghinezhad, & Moghimi, 2018; Movagharnejad & Nikzad, 2007; Shekarchizadeh, Tikani, & Kadivar, 2014).

Liu et al. (2019) have surveyed ANNs application to predict *E_u_*, *EUR*, exergy loss, and exergy efficiency mushroom slices in HA dryer. The results indicated that value *R* ^2^ for *E_u_*, *EUR*, exergy loss, and exergy efficiency was .9978, .985, .994, and .998, respectively. Checking drying index (moisture content (*MC*) and drying rate) and thermodynamic parameters (energy and exergy efficiency) of drying banana in HA flow dryer with the help combined structure ANNs‐RSM showed that this structure is able to predict drying index and thermodynamic parameters with *R* ^2^ > .96 and RMSE < 0.060 (Taheri‐Garavand, Karimi, Karimi, Lotfi, & Khoobbakht, 2018). Kaveh, Jahanbakhshi, et al. (2018) tried to predict *MR* of almond fruit in the process of drying in a convective dryer with ultrasound pretreatment through mathematical models, ANNs and ANFIS and reported that to predict moisture ratio in almond, ANFIS model with the *R* ^2^ = .9998 and *MSE* = 0.003 had a better performance.

It was founded in a study in which potato slice, energy and exergy in fluidized bed method was modeled with the help of the neural network that can predict energy and exergy of potato slice with best and highest accuracy. In this investigation, drying time, air temperature, inlet airspeed, and depth variables were considered as network inputs (Azadbakht, Aghili, Ziaratban, & Torshizi, 2017).

The detailed literature review for the present study has shown that there is no information on energy and exergy analysis and other parameters of the thin‐layer drying process of quince fruit via hot air dryer. Therefore, this paper, as a novel study, concentrates on the energy and exergy analysis of the thin‐layer drying of quince fruit via hot air dryer by using the first and second law of thermodynamics. The primary objective of this study is to present modeling, analysis of kinetics, *D_eff_*, *E_a_*, *SEC*, energy and exergy analysis of thin‐layer drying of quince fruit at different conditions in a hot air dryer. It is believed that such a study will contribute to quince fruit producers by removing their problems related to energy and exergy throughout the drying process.

## 2 MATERIALS AND METHODS

### 2.1 Preparing the samples

In this research, quince fruits were purchased from a local market in Ardebil, Iran**.** After being washed, fruits in good shape were selected for the experiment. Samples were stored in a refrigerator at the temperature of 4°C to be prepared and to reach similar initial temperature before the experiments. The initial *MC* of the samples was obtained by using the Memmert standard oven for 24 hr at the temperature of 70°C (Jahanbakhshi, 2018), which was equal to 5.52 ± 0.5% on a dry basis.

### 2.2 Hot air dryer

To carry out the experiments, a HA dryer was used. The samples to be tested were placed in the middle of the airflow channel of the dryer in a meshed bowl on a digital scale with a precision of 0.01 g attached below the dryer and situated outside the airflow channel. The laboratory dryer used in this study had a centrifugal blower, which blew HA over the samples in a parallel manner. To begin the experiment, the device was first put on and worked for 15 min so that the dryer would reach balance temperature. Then, the quince samples were placed on the dryer's bed and the weight of the samples was measured in 5‐min intervals by a scale (AND, GF‐6000) and recorded in a computer.

The relative temperature and moisture in the ambient around the dryer are two determining variables in drying foodstuff. Thus, in each experiment, the relative temperature and moisture around the dryer were measured and recorded using a digital Testo 925 thermometer with the accuracy of ±0.1°C and a Testo 400 moisture meter with the accuracy of ±0.1%.

During the drying experiments, the average ranges for changes in ambient temperature and relative air moisture were 25 ± 4°C and 17 ± 5%, respectively. The experiments were conducted at temperatures (50, 60, and 70°C) and three input air velocities (0.6, 1.2, and 1.8 m/s). The thickness of 3 mm was selected for the samples in this study.

The relative moisture ratio (*MR*) of the quince fruit samples can be obtained through Equation (1) (Jahanbakhshi, Rasooli Sharabiani, Heidarbeigi, Kaveh, & Taghinezhad, 2019; Torki‐Harchegani, Ghanbarian, Pirbalouti, & Sadeghi, 2016).

(1) $$MR\;=\;\frac{M_{t}\;-\;M_{e}}{M_{b}\;-\;M_{e}}$$

### 2.3 Modeling

To model the drying process, the relative *MR* of the quinces in different treatments was calculated using Equation (1). After determining the relative *MR* values, the data were fitted using ten mathematical models (Table 1) in MATLAB R2014a software.

**Table 1:** Applied models to fit the experimental data

Models	Equations	References
Newton (Lewis)	MR=exp(-kt)	Elmas et al. (2019)
Henderson and Pabis	MR=aexp(-kt)	Torki‐Harchegani et al. (2016)
Page	$MR\;=\;\exp\;(-kt^{n})$	Khanali and Rafiee (2014)
Logarithmic	MR=aexp(-kt)+c	Arepally et al. (2017)
Two‐term	$MR\;=\;a\;\exp\;\left(-k_{0}t\right)+b\;\exp\;\left(-k_{1}t\right)$	Ziaforoughi et al. (2016)
Wang and Singh	$MR\;=1\;+\;at\;+\;bt^{2}$	Sahin and Doymaz (2017)
Midilli *et al.*	$MR\;=\;a\exp\;(-kt^{n})\;+\;bt$	Darıcı and Sen (2015)
Parabolic	$MR\;=\;a\;+\;bt\;+\;ct^{2}$	Coskun et al. (2017)
Logistic	MR=a/(1+bexp(kt))	Rad et al. (2018)
Demir et al.	$MR\;=\;a\exp\;{\left(-kt\right)}^{n}\;+\;b$	Kaveh, Jahanbakhshi, et al. (2018)

One of the most important criteria used for specifying the best model is the coefficient of determination (*R* ^2^), and the appropriate fitting can be determined using an index called root mean square error (*RMSE*). The model, which has the highest *R* ^2^ and the lowest *RMSE*, will be the best model for treatment. These values can be calculated using Equations 2 and 3 (Amiri Chayjan, Kaveh, & Khayati, 2017).

(2) $$R^{2}\;=\;1\;-\;\frac{\sum_{i=1}^{N}\;{\left(y\;-\;y^{\prime }\right)}^{2}}{\sum_{i=1}^{N}\;{\left(y\;-\;\tilde{y}\right)}^{2}}$$

(3) $$RMSE\;=\;\sqrt{\frac{1}{N}\;\sum_{i=1}^{N}\;{\left(y\;-\;y^{\prime }\right)}^{2}}$$

### 2.4 Determination of *D_eff_*

Fick's second law is used extensively to describe diffusivity in the process of drying agricultural products (Mohammadi et al., 2019):

(4) $$\frac{\partial M}{\partial t}=D_{\mathrm{eff}}\nabla^{2}\frac{\partial^{2}M}{\partial x^{2}}$$

The initial and boundary conditions are:

(5) $$M\left(t=0,x\right)=M_{o};\frac{\partial M}{\partial x}\left(t,x=0\right)\;\text{and}\;M\left(t,x=\pm \frac{L}{2}\right)=M_{s}$$

After extending Equation (5) and maintaining the drying conditions for a long time, Equation (6) can be obtained for determining moisture diffusivity (Koukouch et al., 2017):

(6) $$MR=\frac{M_{t}-M_{e}}{M_{o}-M_{e}}=\frac{8}{\pi_{2}}\sum_{n=1}^{\infty }\frac{1}{\left(2n+1\right)}\exp\left(\frac{-D_{\mathrm{eff}}{\left(2n+1\right)}_{2}\pi^{2}t}{4L^{2}}\right)$$

Effective moisture diffusivity coefficient (*D_eff_*) is obtained through Equation (7) from the gradient (*K*) of the *Ln* (*MR*) graph over time:

(7) $$K=\left(\frac{D_{\mathrm{eff}}\pi^{2}}{4L^{2}}\right)$$

### 2.5 Determination of *E_a_*

Using the Arrhenius equation, the relationship between temperature and *D_eff_* is obtained and activation energy can be calculated (Mohammadi et al., 2019).

(8) $$D_{\mathrm{eff}}\;=\;D_{0}\;\exp\;\left(\;-\;\frac{E_{a}}{R_{g}T_{a}}\right)$$

By drawing the graph *Ln* (*D_eff_*) in front of (*1/T_a_*), a line with the gradient *K_1_* is obtained:

(9) $$K_{1}\;=\;\left(\frac{E_{a}}{R_{g}}\right)$$

### 2.6 Determination of *SEC*

The value of energy needed to evaporate a kilogram of water from the product in the drying process is defined as *SEC*. Value of the *SEC* used in an HA dryer is taken from two sources. These energies are (a) heat energy (thermal energy) and (b) blower energy (mechanical energy). The heat generator energy is obtained from Equation (10) (Onwude, Hashim, Abdan, Janius, & Chen, 2019):

(10) $$EU_{\mathrm{ter}}=\left(A_{\mathrm{dc}}.V_{a}.C_{\mathrm{pa}}.\rho_{a}.\Delta T.t\right)$$

The value of *ρ_a_* can be obtained by Equation (11) (Motevali, Minaei, Banakar, Ghobadian, & Khoshtaghaza, 2014):

(11) $$\rho_{a}=\frac{101.325}{0.287T}$$

The mechanical energy obtained from the blower is calculated through Equation (12) (Rad, Kaveh, Sharabiani, & Taghinezhad, 2018):

(12) $$EU_{\mathrm{mec}}=\Delta P.M_{\mathrm{air}}.t$$

The *SEC* of the quince fruit in the HA drying is obtained from Equation (13) (Motevali et al., 2014; Rad et al., 2018):

(13) $$SEC=\frac{EU_{\left(mec+ter\right)}}{M_{W}}$$

### 2.7 Energy analysis

The energy utilization (*E_u_*), mass flow of air (*ṁ_da_*), dry air enthalpy (*h_da_*), specific heat of input and output air (*C_pda_*), air humidity ratio (kg water/kg dry air) (*w*), ratio of air humidity to the inlet and outlet (*w*_dao_), mass transfer rate (kg water/s) (*ṁ_v_*), and energy utilization ratio (*EUR*) calculated through the first law of thermodynamics can be expressed as follows (Table 2):

**Table 2:** Formulas used for determining energy utilization and energy utilization ratio of convective dryer

Equation	Equation number	Reference
$Eu\;=\;{\dot{m}}_{\mathrm{da}}\;\left(h_{\mathrm{dai}}\;-\;h_{\mathrm{dao}}\right)$	(14)	Nazghelichi et al. (2010)
${\dot{m}}_{\mathrm{da}}\;=\;\rho_{a}V_{a}A_{\mathrm{dc}}$	(15)	Khanali and Rafiee (2014)
$h_{\mathrm{da}}\;=\;C_{\mathrm{pda}}\;\left(T\;-\;T_{\mathrm{ref}}\right)\;+\;h_{\mathrm{fg}}w$	(16)	Nazghelichi, Aghbashlo, Kianmehr, and Omid (2011)
$C_{\mathrm{pda}}\;=\;1.004\;+\;1.88w$	(17)	Azadbakht et al. (2017)
$w\;=\;0.622\;\times \;\frac{\phi \;\times \;P_{\mathrm{vs}}}{P\;-\;P_{\mathrm{vs}}}$	(18)	Zohrabi, Seiiedlou, Aghbashlo, Scaar, and Mellmann (2019)
$w_{\mathrm{dao}}\;=\;w{}_{\mathrm{dai}}\;+\;\frac{{\dot{m}}_{v}}{{\dot{m}}_{\mathrm{da}}}$	(19)	Azadbakht et al. (2017)
${\dot{m}}_{v}\;=\;\frac{w_{t}\;-\;w_{t+\Delta t}}{\Delta t}$	(20)	Khanali and Rafiee (2014)
$EUR\;=\;\frac{{\dot{m}}_{\mathrm{da}}\;\left(h_{\mathrm{dai}}\;-\;h_{\mathrm{dao}}\right)}{{\dot{m}}_{\mathrm{da}}\;\left(h_{\mathrm{dai}}\;-\;h_{\mathrm{dae}}\right)}$	(21)	Nazghelichi et al. (2010)

### 2.8 Analysis of exergy

Exergy of air at the entrance to the drying chamber, exergy of air at the outlet of the drying chamber, exergy loss (*Ė x_y_*), and exergy efficiency (*η_Ex_*) obtained using (Equations 21–24) in (Table 3).

**Table 3:** Formulas used for determining Exergy loss and Exergy loss of convective dryer

Equation	Equation number	Reference
$\dot{E}x_{\mathrm{dci}}\;=\;{\dot{m}}_{\mathrm{da}}\;C_{\mathrm{Pda}}\;\left[\left(T_{\mathrm{dci}}\;-\;T_{\infty }\right)\;-\;T_{\infty }\ln\frac{T_{\mathrm{dci}}}{T_{\infty }}\right]$	(22)	Zohrabi et al. (2019)
$\dot{E}x_{\mathrm{dco}}\;=\;{\dot{m}}_{\mathrm{da}}\;C_{\mathrm{Pda}}\;\left[\left(T_{\mathrm{dco}}\;-\;T_{\infty }\right)\;-\;T_{\infty }\ln\frac{T_{\mathrm{dco}}}{T_{\infty }}\right]$	(23)	Motevali et al. (2018)
$\dot{E}x_{y}\;=\;\dot{E}x_{\mathrm{dci}}\;-\;\dot{E}x_{\mathrm{dco}}$	(24)	Arepally et al. (2017)
$\eta_{\mathrm{Ex}}\;=\;\frac{\dot{E}x_{\mathrm{dci}}\;-\;\dot{E}x_{y}}{\dot{E}x_{\mathrm{dci}}}\;=\;1\;-\;\frac{\dot{E}x_{y}}{\dot{E}x_{\mathrm{dci}}}$	(25)	Liu et al. (2019)

### 2.9 Artificial neural networks

**Figure 1:**
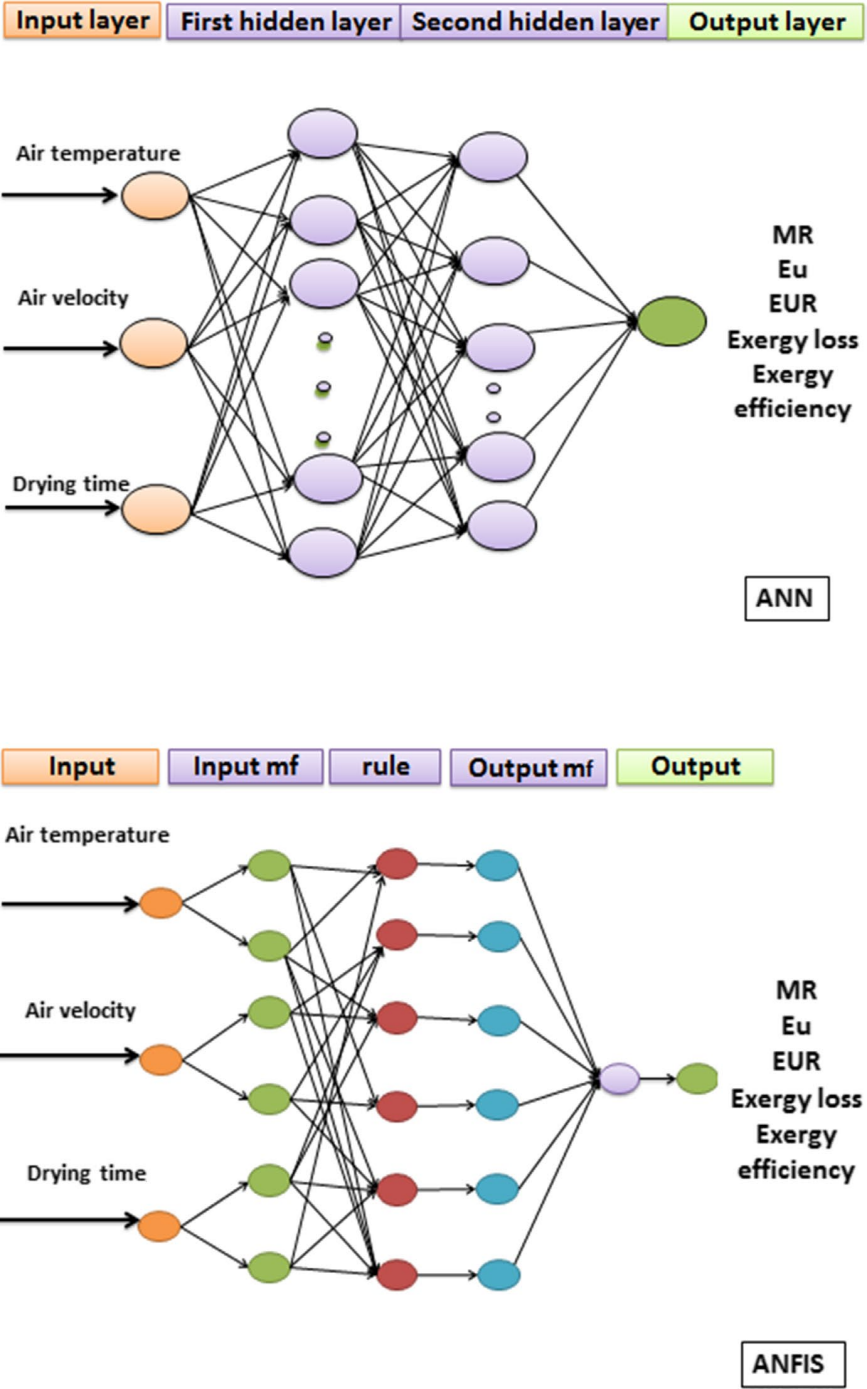
ANN and ANFIS structure

The network architecture consists of an input layer with three neurons, an output layer and one or two hidden layers (Figure 1). The input layer consists of three variables (air temperature, air velocity, and drying time), and the output layer has one variable (*MR, E_u_, EUR,* exergy loss, and exergy efficiency) for the quince fruit drying process. It is difficult to determine the optimal number of neurons in the hidden layer, and it usually depends on the type and complexity of the work. Thus, it is determined by trial and error.

For artificial neural networks, single‐layer and double‐layer cascade Forward Back Propagation (CFBP) and Feed Forward Back Propagation (FFBP) networks were used, with different numbers of neurons varying between 3 and 15. Moreover, the Levenberg‐Marquardt (LM) and Bayesian Regularization (BR) algorithms were used. Three threshold functions namely sigmoid activation function (Logsig), linear activation function (Purelin), and hyperbolic tangent activation function (Tansig) were used to predict the proposed parameters (Jahanbakhshi & Salehi, 2019; Savari, Moghaddam, Amiri, Shanbedi, & Ayub, 2017).

### 2.10 ANFIS

ANFIS consists of a set of if–then rules and pairs of fuzzy input–output data that use artificial neural networks learning algorithms for training (Al‐Mahasneh, Aljarrah, Rababah, & Alu'datt, 2016). ANFIS is very similar to a fuzzy inference system, and the only difference between the two is the use of the backpropagation error algorithm by ANFIS to minimize error. ANFIS's function is very much like those of artificial neural networks and fuzzy logic. In both of these methods, the input passes through the input layer (by the input membership function), and then, the output of the model is obtained in the output layer (by the membership function of the output) (Ziaforoughi, Yousefi, & Razavi, 2016).

Each ANFIS model consists of five layers: (a( Fuzzification, (b( Multiplication, (c( Normalization, (d( Defuzzification, and (e) Summation (Eski et al., 2018) (Figure 1). In the ANFIS analysis, as with other models, the best architecture should be designed. To achieve this objective, ANFIS models are designed using the trial and error method to determine the number of fuzzy rules in output prediction. Eight types of membership functions, namely psigmf, dsigmf, pimf, Gasuss2mf, Gaussmf, gbellmf, trimf, and trapmf can be used as the input to the ANFIS model (Kaveh, Sharabiani, et al., 2018). The number of rules for the membership functions varied from 3 to 5. Moreover, a linear function was selected as the output ANFIS membership function and a hybrid learning method was used.

The present study was modeled using ANNs and ANFIS to predict experimental data on quince fruit drying (*MR, E_u_, EUR,* exergy loss, and exergy efficiency). Training data and data simulation were conducted in Toolbox of Matlab R2014a software. For network modeling, the data were randomly assigned to two groups, of which 75% were used for training and 25% were applied for testing models.

## 3 RESULTS AND DISCUSSION

### 3.1 Drying kinetic

**Figure 2:**
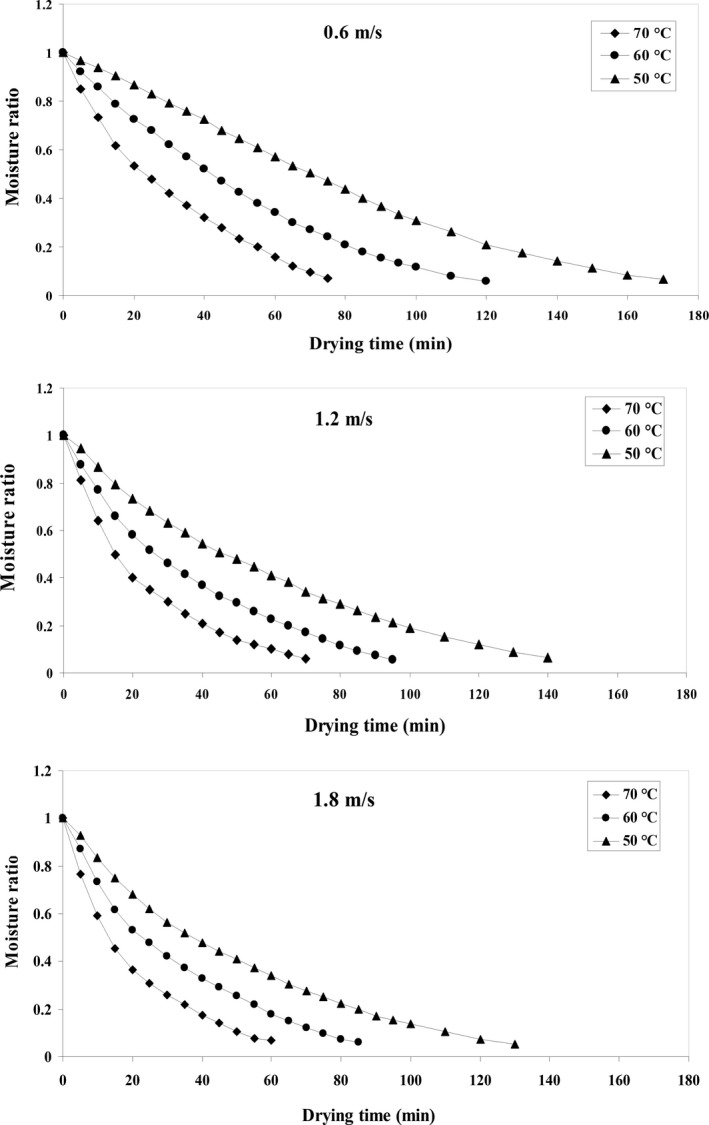
Moisture ratio variation in quince fruit under convective drying (air velocity and air temperature)

After obtaining *MR* at different temperatures and different drying rates, the drying curves were fitted to the experimental data (Figure 2). The results show that the initial *MC* of the product is high and the moisture loss rate is high at the beginning of the drying process (Sahin & Doymaz, 2017). Gradually, as time passes, the initial *MC* of the product decreases naturally over time. The product's drying curve moves downwards at a high gradient at the beginning of the process due to the evaporation of surface moisture, and after this time, due to the onset of water penetration from inside the material to the surface, the curve falls at a lower gradient level (Coskun, Doymaz, Tunçkal, & Erdogan, 2017). At lower velocities, the total drying time is longer. As the temperature rises, the time required for the product to dry decreases due to increased moisture evaporation rate. The effect of temperature on drying time is greater than that of the dryer air velocity in the process of drying the product (Darıcı & Sen, 2015). Moreover, the process of decrease in *MC* under different test conditions shows that increase in air flow rate in the HA dryer reduces the drying time of the product. The reason for this phenomenon is that by increasing air velocity, the vapor pressure of the environment is reduced and, as a result, the *MC* of the product will face less resistance to exit and will be released more quickly (Elmas, Varhan, & Koç, 2019; Kaveh, Sharabiani, et al., 2018). The decreasing of drying time with increasing of drying air temperature and air velocity has been reported for many agricultural products such as jujube slices (Elmas et al., 2019), potato slice, garlic, cantaloupe (Kaveh, Sharabiani, et al., 2018), mushroom slices (Ghanbarian, Dastjerdi, & Torki‐Harchegani, 2016), beef (Ahmat, Barka, Aregba, & Bruneau, 2015).

### 3.2 Drying model

The values of *R* ^2^ and *RMSE* related to the data obtained from the experiments (experimental data) and the data from the models (predicted data) for each of the temperatures and input air velocities are reported in Table 4. Of the ten models, the model with the highest *R* ^2^ value and the lowest *RMSE* value was selected as the appropriate model. According to Table 4, the model proposed by Midilli et al. was selected as the best model for describing the quince fruit's drying behavior in a HA dryer with mean *R* ^2^ of .9992 and *RMSE* of 9.1 × 10^−3^.

**Table 4:** The statistical comparison for prediction of thin‐layer drying of quince

Model	*R* ^2^	*RMSE*
Newton (Lewis)	.9970	0.0152
Henderson and Pabis	.9979	0.0122
Page	.9959	0.0182
Logarithmic	.9980	0.0118
Two‐term	.9975	0.0137
Wang and Singh	.9966	0.0169
**Midilli et al.**	**.9992**	**0.0091**
Parabolic	.9985	0.0105
Logestic	.9987	0.0099
Demir et al.	.9947	0.0219

### 3.3 Effective moisture diffusivity

**Figure 3:**
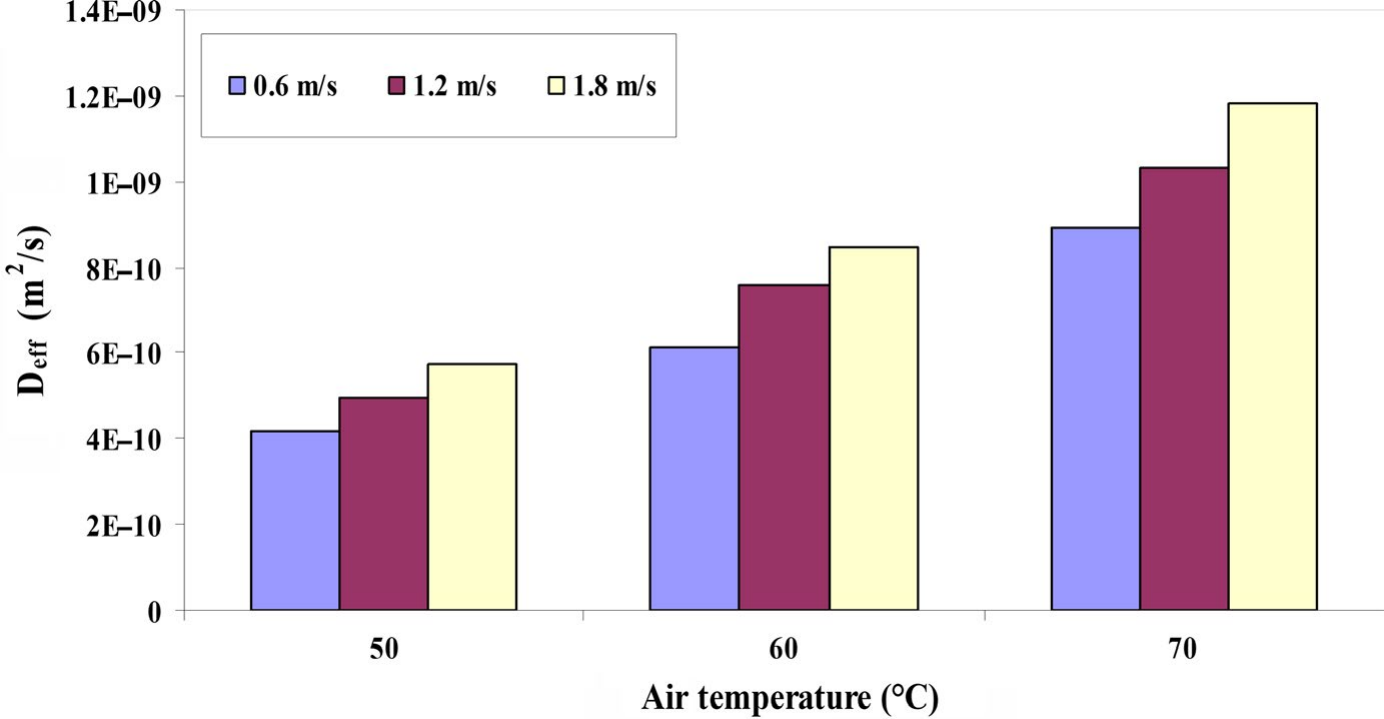
Effect of input air velocity and temperature on effective moisture diffusion coefficient

Figure 3 shows the *D_eff_* of the quince fruit at different temperatures and input air velocities. The results show that the *D_eff_* increases with increase in drying temperature and air velocity. The *D_eff_* varies from 4.19 × 10^−10^ to 1.18 × 10^−9^ for temperature range of 50 to 70°C and air velocity of 0.6 to 1.8 m/s. By increasing the temperature of the dryer chamber, the moisture transfer rate from inside the fruit to its surface increases and the *D_eff_* increases around three times (Sahin & Doymaz, 2017). In addition, the *D_eff_* of quince fruit indicates that the values obtained in this study were within the range of *D_eff_* content for foodstuff which is 10^−12^ to 10^−8^ (Kaveh, Jahanbakhshi, et al., 2018). Similar results about the *D_eff_* have been reported by other researchers that shown in Table 5.

**Table 5:** Effective moisture diffusivity values for some agricultural Products

Fruit	*D*_eff_	Reference
Kiwi	1.94 × 10^–9^ − 7.12 × 10^−9^ m^2^/s	Mohammadi et al. (2019)
Potato	7.84 × 10^–10^ − 2.88 × 10^−9^ m^2^/s	Boutelba et al. (2019)
Mango cubes	1.04 × 10^–8^ − 1.89 × 10^−8^ m^2^/s	Sehrawat, Nema, and Kaur (2018)
Olive‐tree pruning	3.41 × 10^–8^ − 32.5 × 10^−8^ m^2^/s	Cuevas et al. (2019)
Walnut	2.77 × 10^–9^ − 5.56 × 10^−9^ m^2^/s	Abbaspour‐Gilandeh, Kaveh, and Jahanbakhshi (2019)

### 3.4 Activation energy

The value of the *E_a_* for the quince fruit in a HA dryer was obtained within the range of 33.06 to 34.77 kJ/mol (Table 6). Therefore, the amounts reported in this study for the *E_a_* of the quince fruit were within the recommended range (12.7 to 110 kJ/mol) for agricultural products (Aral & Bese, 2016; Samimi‐Akhijahani & Arabhosseini, 2018).

**Table 6:** Activation energy values and related correlation coefficient for quince fruit

Parameter	0.6 m/s	1.2 m/s	1.8 m/s
Activation energy (*E_a_*) (kJ/mol)	34.77	33.71	33.06
Coefficient of determination (*R* ^2^)	.9998	.9954	.9991

Value of the *E_a_* represents value of energy required to start mass transfer from the body of the product. If the *MC* is absorbed by the surface, it requires less energy to start evaporation (Aghbashlo, Kianmehr, & Samimi‐Akhijahani, 2008). In similar studies, *E_a_* for hawthorn was obtained between 78.74 and 91.54 kJ/mol (Aral & Bese, 2016) and for tomatoes, it was 12.43 kJ/mol (Coskun et al., 2017).

### 3.5 Specific energy consumption

**Figure 4:**
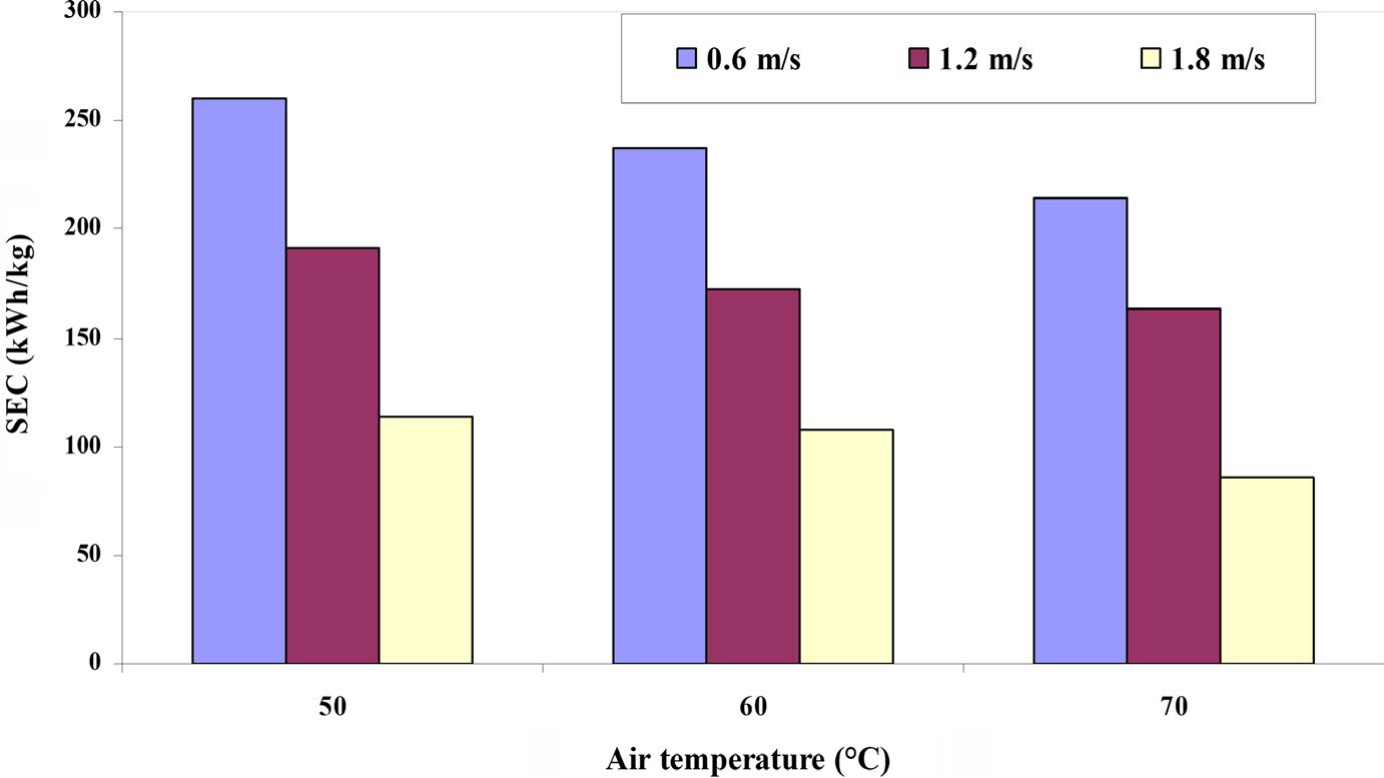
Specific energy consumption over temperature and input air velocity

The maximum and minimum amounts of *SEC* by quince fruit were calculated to be between 85.40 and 260.11 kWh/kg (Figure 4). As the temperature of the input air increased, *SEC* decreased due to the significant increase in the drying rate at higher levels of input air temperature. In other words, although by increase in the temperature of the input air, the thermal power applied increases according to Equation 12, due to the reduction in the drying time, thermal energy required to remove the unit of moisture from the product decreased. At low temperatures, drying time and *SEC* amount increase (Motevali et al., 2014).

Motevali and Tabatabaei (2017) have achieved similar results in examining the drying process for dog‐rose in a HA dryer. For drying apples in a HA dryer, Majdi, Esfahania, and Mohebbi (2019) obtained an *SEC* amount between 5.5 and 8.9 kWh.

### 3.6 Energy utilization

**Figure 5:**
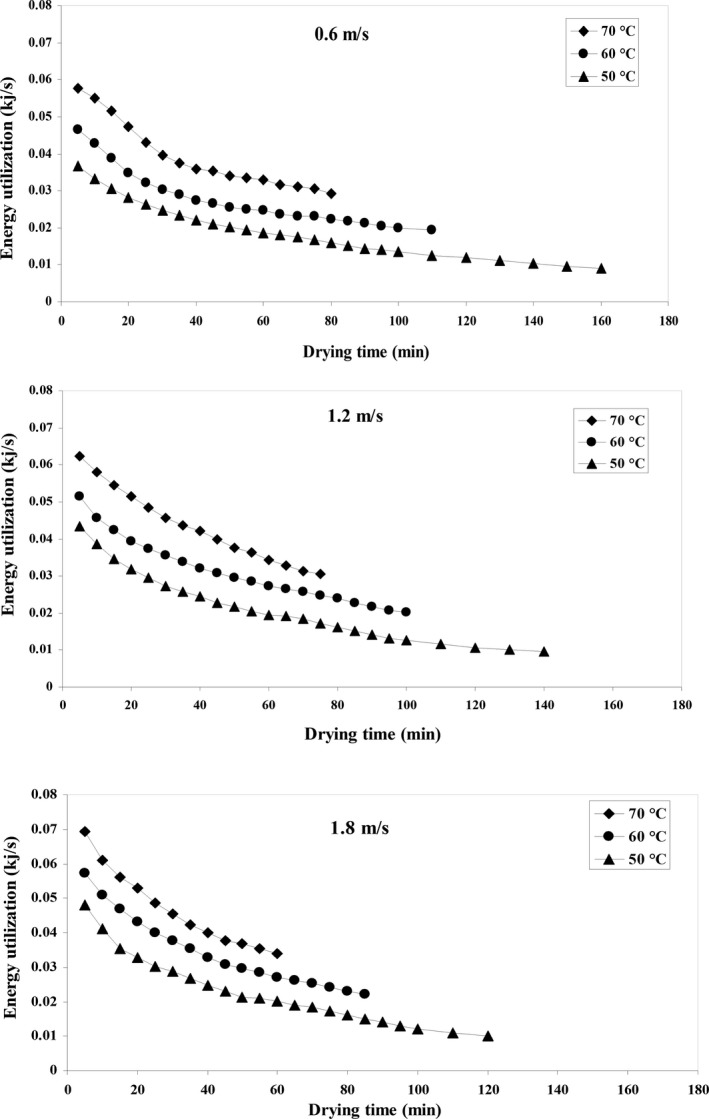
Energy utilization variations against drying time at different air temperature and airflow velocities

Energy utilization analysis for the quince fruit was carried out using data from experiments in a hot air dryer. Figure 5 shows the effect of two parameters of temperature and input air velocity on *E_u_* in the process of drying quince fruit. The maximum of *E_u_* was 0.0694 kJ/s at 70°C and the input air velocity of 1.8 m/s. The minimum amount of *E_u_* was equal to 0.009 kJ/s.

#### 3.6.1 Effect of air temperature

Figure 5 shows that the *E_u_* increased by an increase in the input air temperature. The highest amount of *E_u_* was observed at the beginning of the drying process and this amount decreased with time. During the drying time, due to the faster transfer of moisture, *E_u_* was higher at the beginning of the drying process. Increasing the temperature of the dryer air led to increase in the input enthalpy and higher heat and mass transfer resulting in higher energy consumption and a higher amount of moisture was taken from the product (Nazghelichi, Kianmehr, & Aghbashlo, 2010). These results are similar to those obtained by Azadbakht et al. (2017) for drying a thin layer of potatoes in a fluidized bed dryer, and Aghbashlo, Kianmehr, and Arabhosseini (2008), for drying potatoes in a semi‐industrial continuous dryer.

#### 3.6.2 Effect of air velocity

According to Figure 5, *E_u_* increases with increasing air that enters the drying chamber. Since, the specific energy consumption depends on the velocity of the input air, the hidden heat of water vapor, and the specific heat and the output air temperature, the air mass flow and the enthalpy volume of the input air will increase by increasing airspeed. These conditions will cause the *MC* of the product to evaporate rapidly and increase *E_u_*. These results are consistent with the results of Nazghelichi et al. (2010) regarding drying carrots in the fluidized bed dryer.

### 3.7 Energy utilization ratio

**Figure 6:**
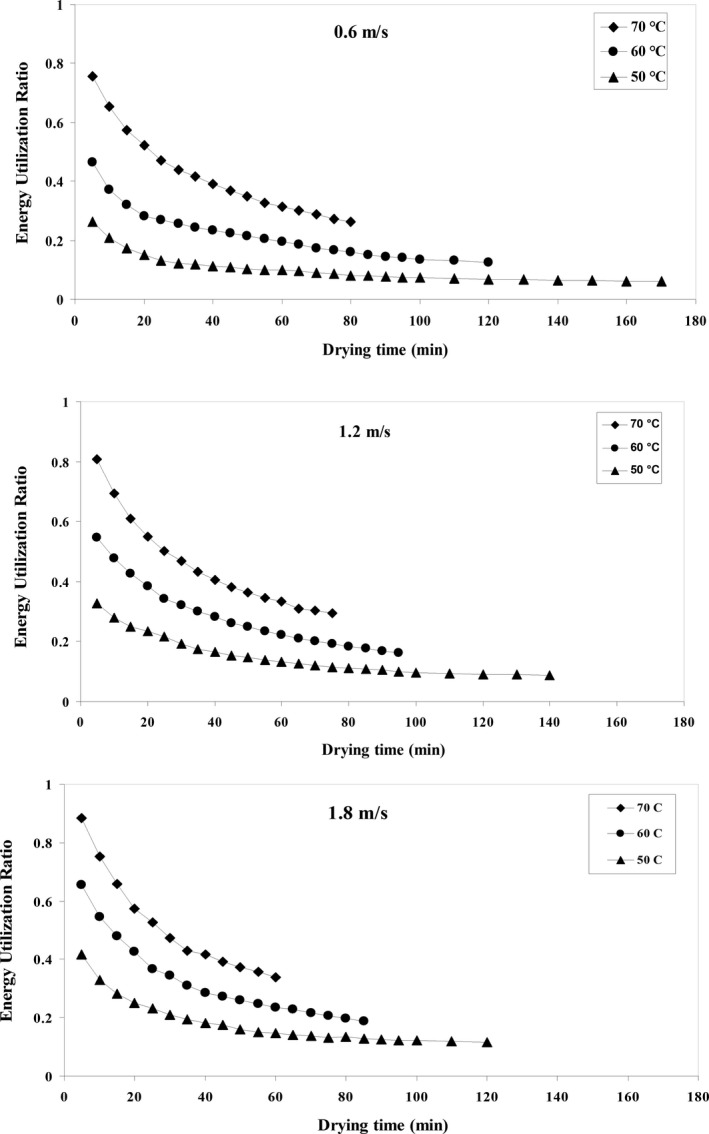
Energy utilization ratio variations against drying time at different air temperature and airflow velocities

Figure 6 shows the *EUR* for the parameters of temperature and air velocity. The highest obtained *EUR* was 0.882 at the temperature of 70°C and airspeed of 1.8 m/s, while the lowest *EUR* was 0.061 at the temperature of 40°C and the airspeed of 0.6 m/s.

#### 3.7.1 Effect of temperature

The results of Figure 6 show that the *EUR* increases by increasing the temperature of the dryer chamber from 50 to 70^ο^C. The highest *EUR* was observed at the beginning of the drying process, after which *EUR* decreased over time. According to the results, the *EUR* increases with increasing wall temperature in the hot air dryer because increasing the temperature of the dryer chamber increases the heat transfer between the dryer's walls and thus increases the evaporation rate of the *MC* of the product (Darvishi, Azadbakht, & Noralahi, 2018). Aviara, Onuoha, Falola, and Igbeka (2014) showed that in a tray dryer used to dry native cassava starch, *EUR* increased by increasing air temperature. Nikbakht, Motevali, and Minaei (2014) reported the use of hot air with microwave pretreatment for drying pomegranate seeds and concluded that increasing the temperature of the input air would increase the *EUR*.

#### 3.7.2 Effect of air velocity

The results show that the *EUR* increases by increasing airspeed. Besides, increasing air velocity increases the removal of moisture from the surface of solid material, which in turn would lead to an increase in the *EUR* in the hot air dryer wall (Yogendrasasidhar & Setty, 2018). Yogendrasasidhar and Setty (2018) have studied on energy and exergy analysis of kodo millet grains and fenugreek seeds in fluidized bed dryer and showed that *EUR* increased with increasing air temperature from 40 to 60°C and airspeed from 1.01 to 1.7 m/s.

### 3.8 Exergy loss

**Figure 7:**
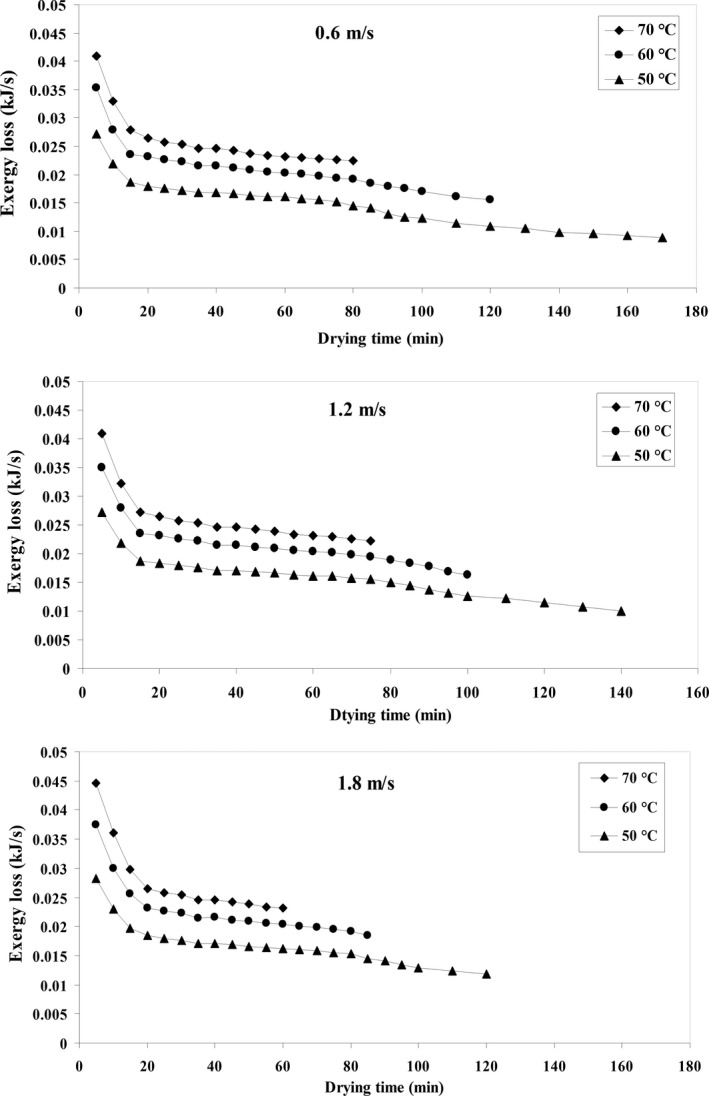
Exergy loss (kJ/s) variations against drying time at different air temperature and airflow velocities

The effects of two parameters of air temperature and input airspeeds on the exergy loss of drying quince fruits in an HA dryer were studied and its results are shown in Figure 7. The highest exergy loss for drying quince fruits was 0.044 kJ/s at the temperature of 70°C and airspeed of 1.8 m/s, and the lowest exergy loss was 0.0088 kJ/s at the temperature of 50°C and the airspeed of 0.6 m/s.

#### 3.8.1 Effect of temperature

To calculate the input exergy, the air temperature of the dryer wall is taken into account (Equation 22) and output exergy is determined by the temperature of the output air (Equation 23). The results for exergy loss are shown in Figure 7. According to Figure 7, exergy loss raised by an increase in air temperature. Exergy loss is higher at the initial drying stage and decreases with drying time. In the initial drying phase, exergy loss is high due to the greater evaporation of the product water. This can be attributed to the fact that the difference between the input and output temperature of the drying chamber is high initially and thus more water evaporation from the product takes place (Darvishi et al., 2018). As time passes, the driving force of mass and moisture transfer decreases and the speed of increase in quince fruit exergy decreases over time, which results more significant exergy loss during the process (Corzo, Bracho, Vasquez, & Pereira, 2008).

Aghbashlo, Kianmehr, and Arabhosseini (2008) conducted an exergy loss study on potatoes in a continuous semi‐industrial dryer and concluded that exergy loss raised with rising air temperature. The maximum exergy loss was reported to be 13.71 kJ/s at 70°C, and the minimum exergy loss was reported with a rate of 0.5987 kJ/s at 50°C. Colak, Kuzgunkaya, and Hepbasli (2008) reported that exergy loss rises from about 0.09 to about 0.12 kJ/s by increasing air temperature from 40 to 50°C for mint leaves in a tray dryer. In another study by Motevali and Minaei (2012) reported the maximum and minimum exergy loss for pomegranate values of 0.1090 and 0.0336 kJ/s, respectively, for an HA dryer with microwave pretreatment. They also reported that by rising air temperature (from 50 to 70°C), the exergy loss rate increases.

#### 3.8.2 Effect of air velocity

To determine the exergy loss, the experiments were performed in the velocity range of 0.6 to 1.8 m/s. The results shown in Figure 7 indicate that the exergy loss decreases by rising airspeed due to the high mass propagation force and it also decreases with the passage of the drying time (Azadbakht, Torshizi, Aghili, & Ziaratban, 2018). Nikbakht et al. (2014) have conducted a study on thermodynamic analysis of pomegranate arils in an HA dryer with microwave treatment and reported that exergy loss increased from 0.0235 to 0.1573 kJ/s with increasing airspeed from 0.5 to 1.5 m/s which is in agreement with the present study where exergy loss increased (for quince fruit 0.0088 to 0.044 kJ/s) with increase in air velocity Hence, the exergy loss rose by rising the air velocity, being in line with the results of Erbay and Icier (2011) on the olive leaves by exergy analysis in a tray dryer.

### 3.9 Exergy efficiency

Exergy efficiency was obtained for quince fruit in the drying experiments at different temperatures and air velocities. The highest amount of exergy efficiency was about 0.879 at the temperature of 70°C and an air velocity of 1.8 m/s. The lowest exergy efficiency was approximately 0.344 at the temperature of 50°C and an air velocity of 0.6 m/s.

#### 3.9.1 Effect of temperature

**Figure 8:**
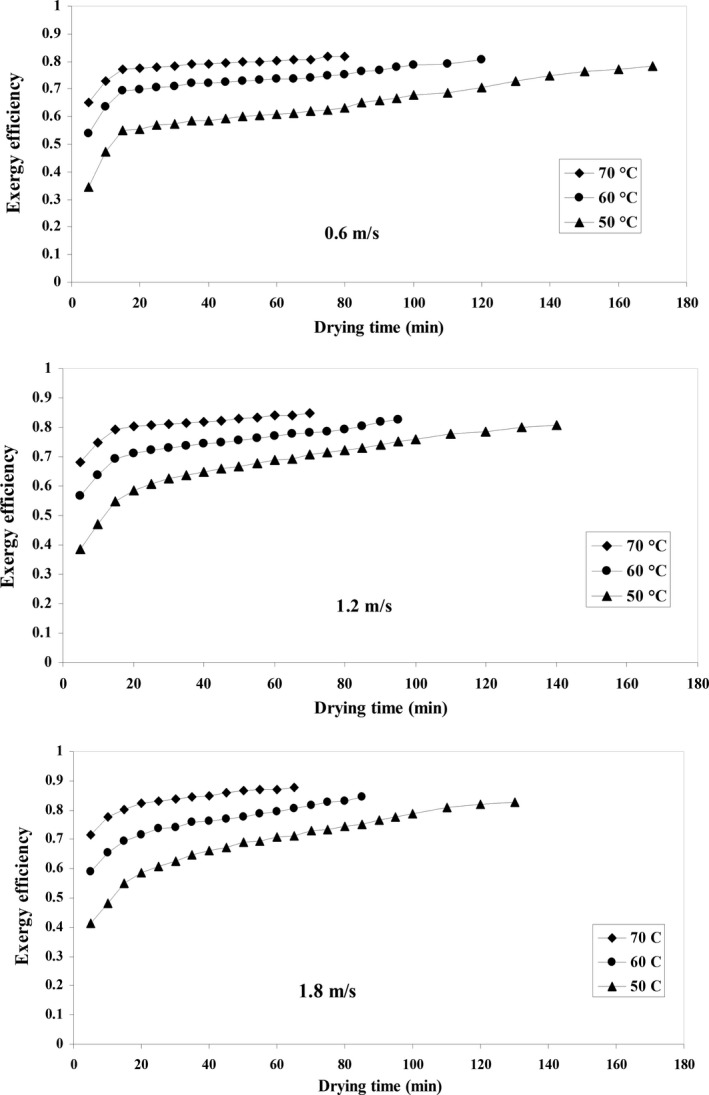
Exergy efficiency variations against drying time at different air temperature and airflow velocities

Exergy efficiency in an HA dryer within the temperature range of 50 to 70°C is shown in Figure 8. It can be seen that exergy efficiency increases by rising air temperature and drying time. Exergy efficiency clearly determines the output exergy of the dryer system in such a way that the output exergy reduces the exergy efficiency due to its high amount of loss in the output air. Exergy loss occurs when the bordering temperature in the dryer is higher than ambient temperature. According to the theory of thermodynamics, exergy efficiency is a proper measure of a drying system (Corzo et al., 2008). These results are similar to what Aktas, Khanlari, Amini, and Sevik (2017) and Ranjbaran and Zare (2013) found about a dryer with infrared‐heat pump on carrot and about drying soybeans in microwave‐assisted fluidized bed dryer, respectively.

#### 3.9.2 Effect of air velocity

Exergy efficiency increases by raising the air velocity (Figure 8). This increase in exergy efficiency can be attributed to the high rate of heating and rapid evaporation that takes place in the quince fruit. The entropy and enthalpy of the input air also increase by raising the input air velocity, which results in raised exergy efficiency (Akpinar, Midilli, & Bicer, 2005). In similar studies, researchers reported that exergy efficiency increased by raising the input airspeed (Akpinar et al., 2005; Azadbakht et al., 2017; Yogendrasasidhar & Setty, 2018).

The dryer's thermodynamic efficiency can be improved by the insulation of the drying chamber, designing and choosing the right components, and selecting optimum drying conditions, using other drying techniques such as microwave, infrared, etc. Exergy efficiency is a valuable tool for detecting key system losses and the optimal performance of industrial dryers.

### 3.10 Artificial neural networks

In this study, a multilayer perceptron (MLP) was used to predict *MR*, *E_u_*, and *EUR* as well as exergy loss and exergy efficiency. Air velocity, temperature, and drying time were considered as inputs of the network. Moreover, kinetic parameters of drying, *E_u_*, and *EUR* as well as exergy loss and exergy efficiency were selected as network output. The training of the network was based on the two algorithms of BR and LM. Before data entering to the network, they were normalized between 0 and 1.

#### 3.10.1 Statistical analysis using neural network

The results of modeling artificial neural networks for predicting (a) *MR*, (b) *E_u_*, (c) *EUR*, (d) exergy loss, and (e) exergy efficiency are reported in Table 7.

**Table 7:** ANN result for *MR*, *E_u_*, *EUR*, exergy loss, and exergy efficiency

Parameter	Network	Training algorithm	Threshold function	Number of layers and neurons	*RMSE*	*R* ^2^	Epochs
Moisture ratio (*MR*)	FFBP	LM	tansig‐ tansig‐ tansig	3‐12‐12‐1	0.0016	.9993	122
Energy utilization (Eu)	FFBP	LM	tansig‐logsig‐ tansig	3‐10‐10‐1	0.0037	.9985	97
Energy utilization ratio (EUR)	FFBP	LM	tansig‐logsig‐purlin	3‐20‐15‐1	0.0029	.9977	65
Exergy loss	CFBP	BR	tansig‐tansig‐tansig	3‐8‐8‐1	0.0032	.9980	142
Exergy efficiency	FFBP	LM	tansig‐logsig‐logsig	3‐10‐10‐1	0.0047	.9970	115

1) To predict the *MR* of the FFBP network, with the topology 3‐12‐12‐1, the tansig–tansig–tansig transfer functions and the LM training algorithm with *R* ^2^ = .9993 and *RMSE* = 0.0016, had the best performance. The results indicate that the ANNs modeling technique can be used effectively to predict *MR*. Similar results have been reported by other researchers for other products such as almond (Kaveh, Jahanbakhshi, et al., 2018), onion (Jafari, Ganje, Dehnad, & Ghanbari, 2016), and mushroom (Omari, Behroozi‐Khazaei, & Sharifian, 2018).

2) ANNs modeling results for predicting *E_u_* during the process of drying quince fruits are given in Table 7. Given the estimated value of *RMSE* = 0.0037 and *R* ^2^ = .9985, the best FFBP network has a topology of 3‐10‐10‐1, with the tansig–logsig–tansig transfer functions and LM training algorithm. Similar results have been reported to predict *E_u_* by other authors including Nikbakht et al. (2014) for pomegranate arils and Azadbakht et al. (2017) for potato cubes using artificial neural networks.

3) According to Table 4, the FFBP network, with 20 neurons in the first hidden layer and 15 neurons in the second hidden layer, with the tansig–logsig–purlin transfer function, as well as the LM algorithm, had the best performance in predicting the *EUR*. In addition, in this network, *RMSE* = 0.0029 and *R* ^2^ = .9977. Nikbakht et al. (2014) used ANNs to predict the *EUR*. They obtained a value of *R* ^2^ = .9680.

4) The CFBP network with BR algorithm, the tansig–tansig–tansig transfer function, with eight neurons in the first and second hidden layers, and *RMSE* = 0.0032 and *R* ^2^ = .9980, had the best prediction for exergy loss.

5) The results of ANNs prediction for exergy efficiency in drying quince fruit in a HA dryer showed that the FFBP network, with a topology 3‐10‐10‐1, the transfer function of tansig–logsig–logsig and the LM training algorithm with *RMSE* = 0.0047 and *R* ^2^ = .9970, was selected as the best network. Taheri‐Garavand et al. (2018) used ANNs to predict exergy efficiency in drying banana slices in a forced convective dryer. They reported a value of *R* ^2^ = .9902. Aghbashlo, Mobli, Rafiee, and Madadlou (2012) also predicted exergy efficiency using artificial neural networks for fish oil and skim milk powder. They obtained *R* ^2^ = .9994 and *MSE* = 7.79 × 10^−5^.

### 3.11 ANFIS

Some changes are required in order to find the best ANFIS network for predicting *MR*, *E_u_*, *EUR*, exergy loss, and exergy efficiency. These changes include the type of membership function, the number of membership functions, and the number of cycles.

The best results for predicting the parameters in question are shown in Table 8. The best MF to predict each of the five parameters after the various changes in the type of membership function was gaussmf membership function. The number of the MFs was determined by the number of the inputs of each ANFIS model.

**Table 8:** ANFIS result for *MR*, *E_u_*, *EUR*, exergy loss, and exergy efficiency

Parameters	Type of MF		Number of MF		Learning method	*RMSE*	*R* ^2^
	Input	Output	Input	Cycle			
Moisture ratio (*MR*)	Gaussmf	Linear	3‐3‐3	1,200	Hybrid	0.0011	.9997
Energy utilization (Eu)	Gaussmf	Linear	3‐3‐3	1,200	Hybrid	0.0028	.9989
Energy utilization ratio (*EUR*)	Gaussmf	Linear	3‐5‐3	1,200	Hybrid	0.0022	.9988
Exergy loss	Gaussmf	Linear	3‐3‐3	1,200	Hybrid	0.0030	.9986
Exergy efficiency	Gaussmf	Linear	3‐5‐3	1,200	Hybrid	0.0046	.9978

According to Table 2, 8, *R* ^2^ for *MR*, *E_u_*, *EUR*, exergy loss, and exergy efficiency was .9997, .9989, .9988, .9986, .9978, respectively. Also, the values *RSME* for *MR*, *E_u_*, *EUR*, exergy loss, and exergy efficiency were 0.0011, 0.0028, 0.0022, 0.0030, and 0.0046, respectively.

### 3.12 Comparison between ANNs and ANFIS model

Comparison of statistical parameters between ANFIS and ANNs models confirms that the ANFIS model has a more accurate performance for predicting each of the five parameters (*MR*, *E_u_*, *EUR*, exergy loss, and exergy efficiency). According to Tables 7 and 8, it can be seen that the value of *R* ^2^ for each of the five parameters predicted in the ANFIS model was higher than the artificial neural networks model. Moreover, the value for the ANFIS in all the predicted parameters was lower than the artificial neural networks model.

Khoshnevisan, Rafiee, Omid, and Mousazadeh (2014) used artificial neural networks and ANFIS models to predict the performance of potatoes. They reported that the ANFIS model performed better than the artificial neural network model with respect to its higher *R* ^2^ and lower *MSE*. Kaveh, Sharabiani, et al. (2018) predicted four parameters (*D_eff_*, *SEC*, *MR*, and *DR*) in drying potato, garlic, and cantaloupe using HA dryers. The ANFIS model showed a higher ability to predict these parameters than artificial neural networks.

## 4 CONCLUSIONS

In this research, the effect of temperature and air velocity on drying time, *D_eff_*, *SEC*, *E_u_*, *EUR*, exergy loss, and exergy efficiency was investigated. The results of the research showed that among the experimental models, the model of Midilli et al. to describe the kinetics of drying the thin layer of quince fruit slices can be introduced as the most suitable model. The range of *D_eff_* in quince fruit samples varied from 4.19 × 10^−10^ to 1.18 × 10^−9^ m^2^/s, regardless of shrinkage, in the range of temperatures and velocities studied. The highest value of *D_eff_* was obtained at the highest levels of temperature and inlet air velocity. With increasing air temperature and air velocity, specific energy consumption, energy utilization, energy utilization ratio, exergy loss, and exergy efficiency increased. The highest *E_u_* and *EUR* were 0.0694 and 0.882 kJ/s at 70°C, respectively. The lowest *E_u_* and *EUR* were 0.009 and 0.061 kJ/s at 50°C, respectively. The highest exergy losses and exergy efficiencies were calculated as 0.044 and 0.879 kJ/s, respectively. Also, the lowest exergy losses and exergy efficiencies were calculated as 0.0088 and 0.344 kJ/s, respectively. ANFIS was one of the fastest methods compared to artificial neural networks with lower *RMSE* and higher *R* ^2^ for estimating *MR*, *E_u_*, *EUR*, exergy loss, and exergy efficiency for quince fruit. By insulating the drying chamber, designing and selecting the right components, as well as selecting the optimum drying conditions, the thermodynamic efficiency of the hot air dryer can be increased. Exergy efficiency is a valuable tool for identifying key system losses and optimizing the performance of hot air dryers.

## NOMENCLATURE

*A_dc_*: Surface area drying chamber (m^2^)
*C_pa_*: Specific heat (kJ/kg C)
*C_pda_*: Specific heat of inlet and outlet air
*Eu*: Energy utilization (kJ/s)
*EUR*: Energy utilization ratio (dimensionless)
*EU*_mec_: Mechanical energy consumption (Kwh/kg)
*EU_ter_*: Thermal energy consumption (Kwh/kg)
*E*_a_: Activation energy (kJ/mol)
$\dot{E}x_{\mathrm{dci}}$: Exergy inlet air (kJ/s)
$\dot{E}x_{\mathrm{dco}}$: Exergy outlet air (kJ/s)
$\dot{E}x_{y}$: Exergy loss (kJ/s)
*D_0_*: Width from the source, which is a constant value
*D_eff_*: Effective moisture diffusivity coefficient (m^2^/s)
L: Half of the thickness of each sample
*M*_w_: Weight loss in the samples (kg)
*h_fg_*: Latent heat of vaporization of water (kJ/kg)
*h_da_*: Specific enthalpy drying air (kJ/kg)
*h_dai_*: Specific enthalpy of inlet air (kJ/kg dryair)
*h_dao_*: Specific enthalpy of outlet air
*h_dae_*: Specific enthalpy of air environment (kJ/kg dry air)
*C_pda_*: Specific heat drying air (kJ/kg K)
${\dot{m}}_{v}$: Mass transfer rate (kg water/s)
${\dot{m}}_{\mathrm{da}}$: Mass flow rate of drying air (kg/s)
*MR*: Moisture ratio
*M_e_*: Equilibrium moisture content (% d.b.)
*M_t_*: Moisture content (% d.b.)
*M_b_*: Initial moisture content (% d.b.)
*n*: index of a summation and the number of terms taken into consideration
*N*: Number of observations
*P*: Atmospheric pressure (kPa)
*P_vs_*: Saturated pressure (kPa)
*R*^2^: Correlation coefficient
*RMSE*: Root Mean Square Error
*R_g_*: Universal gas constant equal to 8.3143 kJ/mol
*SEC*: Specific energy consumption (kWh/kg)
*t*: Drying time
*T_a_*: Air temperature inside the drying chamber (K)
*T_ref_*: Refers to characteristic value
*T*: Temperature (K)
*T_dci_*: Inlet air temperature of drying chamber (K)
*T_dco_*: Outlet air temperature of drying chamber (K)
$T_{\infty }$: Temperature of outlet air (°C)
*Va*: Input air velocity (m/s)
$w_{t+\Delta t}$: Weight of product at time t + t (kg)
*w*: Humidity ratio of air (kg water/kg drying air)
*w_t_*: Weight of product at time *t* and
*w_dao_*: Outlet humidity ratio of air (kg water/kg drying air
*w_dai_*: Inlet humidity ratio of air (kg water/kg drying air)
*y*: Stands for the experimental values
*y*′: Predicted values by calculating from the model for this measurements
$\tilde{y}$: The average predicted values
Δt: Time between two sample weighing (s)
ϕ: Relative humidity of air
$\rho_{a}$: Air density (kg/m^3^)
ΔP: Pressure difference (mbar)
Δt: Temperature difference (°C)
$\eta_{\mathrm{Ex}}$: exergy efficiency

## CONFLICT OF INTEREST

The authors have declared no conflict of interest.

## ETHICAL APPROVAL

This study does not involve any human or animal testing.
